# Molecular Logic of Prokaryotic Surface Layer Structures

**DOI:** 10.1016/j.tim.2020.09.009

**Published:** 2021-05

**Authors:** Tanmay A.M. Bharat, Andriko von Kügelgen, Vikram Alva

**Affiliations:** 1 Sir William Dunn School of Pathology, University of Oxford, Oxford OX1 3RE, UK; 2 Central Oxford Structural Microscopy and Imaging Centre, University of Oxford, Oxford OX1 3RE, UK; 3 Department of Protein Evolution, Max Planck Institute for Developmental Biology, Max-Planck-Ring 5, Tübingen 72076, Germany

**Keywords:** surface layer, bacterial cell envelopes, protein evolution, bioinformatics, structural biology

## Abstract

Most prokaryotic cells are encased in a surface layer (S-layer) consisting of a paracrystalline array of repeating lattice-forming proteins. S-layer proteins populate a vast and diverse sequence space, performing disparate functions in prokaryotic cells, including cellular defense, cell-shape maintenance, and regulation of import and export of materials. This article highlights recent advances in the understanding of S-layer structure and assembly, made possible by rapidly evolving structural and cell biology methods. We underscore shared assembly principles revealed by recent work and discuss a common molecular framework that may be used to understand the structural organization of S-layer proteins across bacteria and archaea.

## Highlights

Despite enormous sequence diversity in surface (S)-layer proteins, structural diversity is much lower than previously thought.
S-layer proteins have a bipartite arrangement with a lattice-forming and an anchoring segment.
Novel structural biology methods are revealing the architectures of S-layers *in situ.*
S-layer assembly across prokaryotes is tightly coupled to the cell cycle, including the cell division machinery.

## The S-layer Constitutes the Outermost Shell of Most Prokaryotic Cells

All living cells must interact with their environment to obtain nutrients, find ecological niches for growth, and defend against extracellular attack by predators or viruses. Cellular interaction with the environment is shaped by molecules on the cell surface that detect stimuli and mediate an appropriate cellular response. This need for specialized cell-surface molecules is particularly crucial for prokaryotes [1] because these molecules can enable cellular motility, initiate cellular adhesion to surfaces [2], mediate biofilm formation [3], and regulate the transport of molecules.

The outermost surface of most bacteria, and nearly all archaea, is composed of a 2D sheet of repeating surface-layer proteins or glycoproteins, known as an S-layer. Although the function of S-layers in many organisms has not been experimentally verified, S-layers are known to regulate cell shape, co-ordinate cellular contact with the environment, and act as a barrier protecting prokaryotic cells from predators or phages [4,5]. In human pathogens, such as *Clostridium difficile*, *Bacillus anthracis*, and *Campylobacter fetus*, S-layers play a key role in bacterial infections, and in some cases, such as in *B. anthracis*, the loss or disruption of the (Sap) S-layer renders the bacteria completely avirulent in mouse infection models [6., 7., 8., 9.]*.* Due to their high copy numbers, it has been estimated that S-layer proteins, as a group, are one of the most abundant protein families on earth [10].

Due to their striking appearance under the microscope, S-layers have been the subject of inquiry for many eminent structural biologists in the past [11,12]; however, structural and cell biology information on S-layers has been scarce until recently. This is partly due to the lack of suitable methods to study these flexible 2D arrays on cells, and perhaps also due to the absence of S-layers in the common prokaryotic model organisms such as *Escherichia coli* and *Bacillus subtilis*. In the last 3 years there has been renewed interest in the field, with a spate of structural biology, cell biology, and functional genetics studies being published on this topic. In this article we summarize recent work in the light of past information available on S-layers and suggest a molecular framework for understanding S-layer sequence, structure, arrangement, and assembly principles on prokaryotic cells.

## The Sequence Space of Archaeal and Bacterial S-layer Proteins

**Figure 1:**
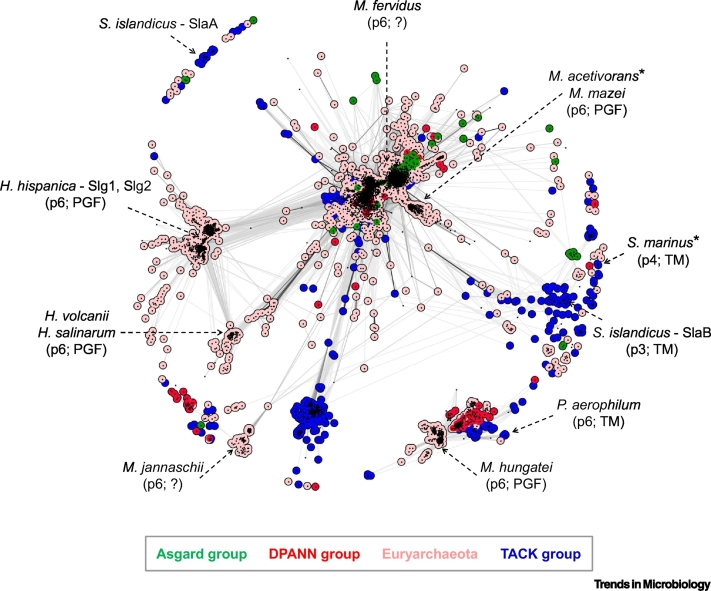
Cluster Map of Archaeal S-layer Proteins. Archaeal S-layer proteins were gathered using BLAST [76] searches and clustered using the CLANS software [77] based on their all-against-all pairwise similarities as measured by BLAST *P* values. Dots represent surface-layer protein (SLP) sequences, and line coloring reflects BLAST *P* values; the darker a line, the lower the *P* value. Colors denote prominent groups of archaea, shown in the box below the map. Some intensely studied archaeal species are highlighted, with the lattice symmetry and predicted anchoring sequence shown in brackets (*denotes S-layers with some structural biology data available). Abbreviations: *H. hispanica*, *Haloarcula hispanica*; *H. salinarum*, *Halobacterium salinarum*; *H. volcanii*, *Haloferax volcanii*; *M. acetivorans*, *Methanosarcina acetivorans*; *M. fervidus*, *Methanothermus fervidus*; *M. hungatei*, *Methanospirillum hungatei*; *M. jannaschii*, *Methanocaldococcus jannaschii*; *P. aerophilum*, *Pyrobaculum aerophilum*; *S. islandicus*, *Sulfolobus islandicus*; *S. marinus*, *Staphylothermus marinus*; TM, transmembrane helix.

**Figure 2:**
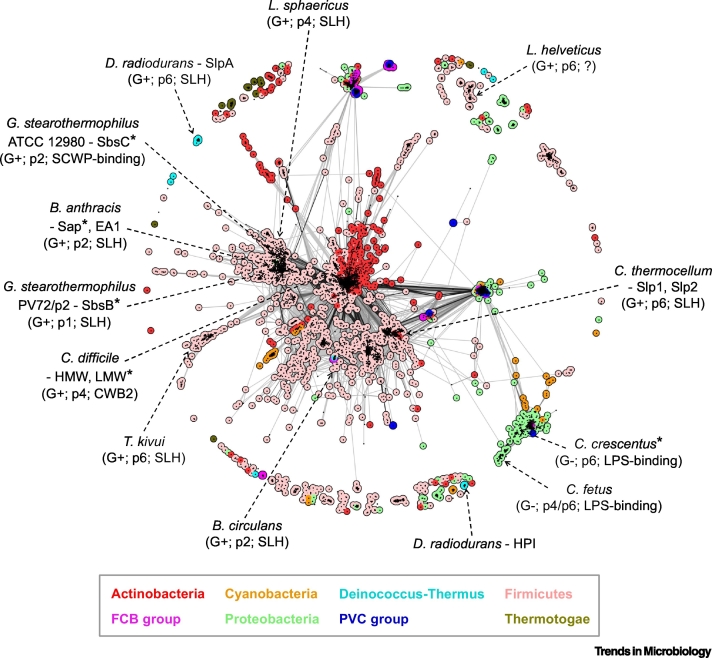
Cluster Map of Bacterial S-layer Proteins. In the same manner as in Figure 1, bacterial S-layer proteins were gathered using BLAST [76] searches and clustered using the CLANS software [77] based on their all-against-all pairwise similarities. Dots represent surface-layer protein (SLP) sequences, and line coloring reflects BLAST *P* values; the darker a line, the lower the *P* value. Colors denote prominent groups of bacteria, shown in the box below the map. Some intensely studied bacterial species are highlighted, with the Gram-type, lattice symmetry, and predicted anchoring sequence shown in brackets (*denotes S-layers with some structural biology data available). Some phyla comprising few representatives in the map, including Acidobacteria*,* Aquificae*,* Chloroflexi, and Spirochaetes, are not colored. Notably, some strains of *C. fetus* use SLPs of different molecular weights to form lattices with p4 or p6 symmetry. Abbreviations: *B. anthracis*, *Bacillus anthracis*; *B. circulans*, *Bacillus circulans*; *C. crescentus*, *Caulobacter crescentus*; *C. fetus*, *Campylobacter fetus*; *C. difficile*, *Clostridium difficile*; *C. thermocellum*, *Clostridium thermocellum*; *D. radiodurans*, *Deinococcus radiodurans*; *G. stearothermophilus*, *Geobacillus stearothermophilus*; *L. helveticus*, *Lactobacillus helveticus*; LPS, lipopolysaccharide; *L. sphaericus*, *Lysinibacillus sphaericus*; SLH, S-layer homology domain; *T. kivui*, *Thermoanaerobacter kivui*.

S-layers are typically composed of a single, or occasionally two or more, species of usually self-assembling extracellular proteins referred to as surface-layer proteins (SLPs) [5,13,14]. These proteins are highly enriched in hydrophobic and acidic amino acid residues and have chain lengths in the range of about 400 to 2500 amino acid residues. Despite similarities in amino acid composition, SLPs exhibit tremendous sequence diversity (Figures 1 and 2) and often share no or minimal similarity at the sequence level, suggesting multiple independent evolutionary origins for them. While sequence similarity between archaeal and bacterial SLPs is rare, high similarity is generally limited to SLPs of closely related organisms (Figures 1 and 2). However, frequently, even SLPs of closely related organisms, such as those of the bacterial genus *Lactobacillus*, display low or undetectable sequence similarities [15]*.*

Despite exhibiting enormous diversity in sequence and length, most hitherto characterized SLPs share a bipartite architecture, comprising a large segment, involved in 2D lattice formation, often in a metal-ion-dependent manner [11,16., 17., 18., 19.], and a smaller segment, involved in anchoring to the cell envelope [20., 21., 22.]. A threonine-rich, intrinsically disordered region separates these two segments in many archaeal SLPs [13,23]. Notably, in some organisms, the lattice-forming and anchoring segments are harbored by two distinct SLPs, for example, in the archaeon *Sulfolobus islandicus* [23]. While the lattice-forming segments are remarkably divergent in their sequences, the anchoring segments show significantly lower sequence variability. They are often even found in SLPs that have otherwise different sequences. Many SLPs of Gram-positive bacteria, for instance, contain three tandem cell-wall-binding S-layer homology (SLH) domains [20].

The comprehensive annotation of the domains of SLPs has largely remained difficult because of their enormous sequence diversity and the lack of homologous domains of known structure. However, the recent structural characterization of many domains of archaeal and bacterial SLPs suggests that their structural diversity is more limited than previously thought. Most characterized SLP lattice-forming segments are built from the repetition and recombination of a limited set of fold types, with a preponderance of immunoglobulin-like β-sandwich, β-roll, β-helical, and coiled-coil folds [7,16,17,24]. The structural diversity exhibited by the anchoring segments is even more limited; they generally are trimers, pseudo-trimers [20,22,25], or bundles of α-rich folds [18,21], or are single membrane-spanning α-helices [23]. Intriguingly, SLPs contain folds found in eukaryotic cell surface and eukaryotic virus envelope proteins, raising exciting questions on the origin of cell-surface proteins across the different domains of life [24,26].

## S-layer Overall Symmetries

S-layers are essentially curved versions of 2D crystals arranged around cells. In general, planar, periodically repeating patterns can be classified according to transformations that leave them invariant, called planar symmetry groups. There are 17 different planar symmetry groups which can be described by translations, rotations, reflections, and glide reflections. Due to limitations imposed by the chiral nature of proteins, including SLPs [27], there are only five general ways of arranging SLPs into a planar crystal with sixfold (p6), fourfold (p4), threefold (p3), twofold (p2)**,** and no rotational symmetry (p1). While hexagonal (p6) symmetries are the most frequently observed arrangement in archaeal and bacterial S-layers, p1, p2, p3**,** and p4 symmetries are also observed [5,13,28,29]. Based on the symmetry type, the unit cells of S-layer lattices are composed of one to six identical subunits, with the center-to-center spacing between the unit cells ranging from 4 to 35 nm [14]. Consequently, the S-layer lattice is heavily punctuated by uniformly distributed pores of identical size and morphology, covering a majority of the total cell surface area. In many S-layers, two or more different classes of pores, with diameters between 2 and 8 nm, have been observed [14].

Many archaea, in particular members of the phylum Euryarchaeota, which comprise the most laboratory-cultured representative species, possess S-layers with a hexagonal lattice (p6) [13]; examples include *Methanocaldococcus jannaschii*, *Methanothermus fervidus*, *Haloferax volcanii*, and *Halobacterium salinarum*. Unlike Euryarchaeota, Crenarchaeota, which are arguably the next best-studied archaeal phylum, do not have a predominant symmetry type and exhibit lattices with p3 (e.g., *S. islandicus*), p4 (*Staphylothermus marinus*), or p6 (*Pyrobaculum aerophilum*) symmetries. S-layer lattice symmetries of most other archaeal phyla, including Lokiarchaeota, Nanoarchaeota, Thaumarchaeota, and Thorarchaeota, remain sparsely characterized; however, some efforts are being made to characterize these elusive organisms [30].

In comparison to archaea, bacterial S-layers do not show a particular preference for hexagonal symmetries, but instead, show a phylogenetically uncorrelated distribution of p1 (e.g., the Gram-positive firmicute *Geobacillus stearothermophilus* PV72/p2), p2 (the Gram-negative betaproteobacterium *Aquaspirillum putridiconchylium),* p4 (the Gram-positive firmicute *Lysinibacillus sphaericus* CCM2177), and p6 (the Gram-negative alphaproteobacterium *Caulobacter crescentus*) symmetries [5,14,29,31,32]. Notably, some bacteria or their different strains (e.g., *C. fetus* and *G. stearothermophilus* strains) display varying lattice symmetries using different SLPs or different molecular weight species of an SLP [33., 34., 35., 36.].

## S-layer Anchoring on Cells

**Figure 3:**
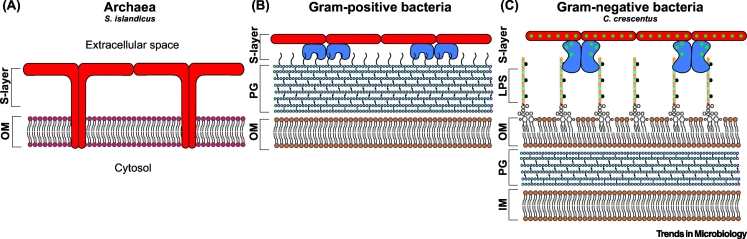
Schematics of Prokaryotic Cell Surfaces. Schematic diagrams of prokaryotic cell surfaces are provided to illustrate the variety of anchoring mechanisms used by S-layers to assemble on cells. (A) Representation of the archaeal cell surface using the crenarchaeon *Sulfolobus islandicus* as an example, experimentally described using high-resolution electron cryotomography (cryo-ET) [23]. Long stalk-like densities of the S-layer are buried within the outer membrane. (B) A generic Gram-positive bacterial outer surface with the S-layer buried within the cell wall. (C) The envelope of the Gram-negative bacterium *Caulobacter crescentus*, as described using cryo-ET [18]. The anchoring domain of the S-layer is noncovalently attached to the O-antigen of LPS. Abbreviations: IM, inner membrane; LPS, lipopolysaccharide; PG, peptidoglycan; OM, outer membrane.

The envelopes of prokaryotic cells are fundamentally different across bacteria and archaea, and therefore S-layers use vastly different mechanisms for anchoring to cell surfaces (Figure 3). In most archaea, S-layers are attached directly to the cytoplasmic membrane [37,38]. In species of the order Methanobacteriales (e.g., *M. fervidus*), however, the cytoplasmic membrane is surrounded by a cell wall composed of pseudopeptidoglycan, also known as pseudomurein, and the S-layer is associated with this pseudomurein layer. While crenarchaeal SLPs, such as those found in the extremophile *S. islandicus*, are generally anchored to the membrane via a C-terminal transmembrane segment [23], most euryarchaeal SLPs appear to be anchored through the covalent attachment of a lipid moiety to their C-terminal end [39., 40., 41., 42.]. Euryarchaeal SLPs typically contain a C-terminally located tripartite segment that comprises a highly conserved PGF (proline-glycine-phenylalanine) motif, a transmembrane helix, and a cluster of basic residues. In *H. volcanii*, this segment is recognized and cleaved by an archaeosortase (ArtA). Furthermore, ArtA also mediates membrane anchoring of the processed SLP through the attachment of a lipid group. Anchoring mechanisms employed by SLPs of pseudomurein-containing archaea remain unclear, but they presumably, like other archaea, also use their C terminus for anchoring.

Compared with archaea, bacteria exhibit more complex cell wall structures. While Gram-positive bacteria have only a thick peptidoglycan layer surrounding the cytoplasmic or inner membrane (IM), Gram-negative bacteria have a thinner peptidoglycan (PG) layer as well as an outer lipid membrane (OM). In many Gram-positive bacteria, SLPs typically contain three – N- or C-terminally located – tandem repeats of the SLH domain which bind noncovalently to PG-linked pyruvylated secondary cell wall polymers (SCWPs) [20,25,43]. For instance, the SLPs of *B. anthracis* (Sap and EA1) each contain three copies each of the SLH domain at their N terminus, whereas those of *Clostridium thermocellum* (SLAP1 and SLAP2) contain three copies each at their C terminus. Unlike most Gram-positive bacteria, in the majority of clostridial species, such as *C. difficile*, *Clostridium tetani, and Clostridium botulinum*, SLPs lack SLH domains and instead contain three tandem repeats of the cell-wall-binding 2 (CWB2) domain, which in *C. difficile* binds to the surface polysaccharide PSII [22,44]. A further anchoring mechanism is exhibited by *G. stearothermophilus* strains, which have five different SLPs, SbsA, SbsB, SbsC, SbsD, and SgsE [5]. While SbsB contains three N-terminal SLH domains, the other four contain three tandem repeats of a third type of SCWP-binding domain. In SbsC, this domain has been shown to interact with a negatively charged SCWP consisting of *N*-acetylglucosamine, glucose, and 2,3-dideoxy-diacetamido mannosamine uronic acid [21]. For some SLPs that lack these anchoring modules, their N or C termini have been implicated in electrostatic interactions with SCWPs. For instance, the C-terminal part of *Lactobacillus acidophilus* ATCC 4356 SlpA is involved in anchoring [45]*,* whereas *Lactobacillus brevis* ATCC 8287 SlpA uses its N-terminal part [46].

Contrary to Gram-positive bacteria, anchoring mechanisms in Gram-negative bacteria remain less well understood. They do not contain any widespread anchoring domains and appear to use their N or C termini to interact with lipopolysaccharides (LPS) in the outer membrane. In the well studied S-layers of *C. crescentus* and *C. fetus*, the N-terminal segments mediate noncovalent interactions with the O-antigen repeating oligosaccharide of the LPS [18,47]. In fact, a recent study characterized the structural basis of the interaction between *C. crescentus* S-layer and the O-antigen oligosaccharide of LPS [18] and showed that newly synthesized SLPs are guided along the LPS to their final position at the tip of the O-antigen.

## Post-translational Modifications of SLPs

In addition to being tremendously diverse in sequence and structure, SLPs display a further level of diversity through post-translational modifications. These modifications are generally species-specific and include cleavage of signal peptides, protein-sorting sequences, or precursor forms; lipidation; glycosylation; and tyrosine phosphorylation [48,49].

Most archaeal and many bacterial SLPs are synthesized with an N- or C-terminal signal peptide (e.g., a bacterial type I secretion signal), a C-terminal sorting signal (e.g., the euryarchaeal PGF-CTERM motif), or occasionally both (e.g., euryarchaeal SLPs typically contain a signal peptide as well as the PGF-CTERM motif). These segments are generally removed following their translocation across the cytoplasmic membrane [5,13,40,41]. Occasionally, in some organisms, the S-layer is formed by the association of two SLPs obtained by the proteolytic processing of a precursor protein [50,51], for instance, in the bacterium *C. difficile* and the archaeon *S. marinus*.

Another widespread post-translational modification observed in many archaeal and bacterial SLPs is the presence of a wide variety of surface-exposed glycans, covalently linked to specific asparagine residues (N-linked glycosylation) or serine/threonine/tyrosine residues (O-linked glycosylation). While both N- and O-linked glycans have been observed in archaeal SLPs, only O-linked glycans have been observed in hitherto characterized bacterial SLPs [5,13,14,52., 53., 54.]. In recent years, glycosylated SLPs, as well as proteins involved in the synthesis, transport, and linkage of the associated glycans, have been characterized in several bacteria (e.g., *G. stearothermophilus*) [35,55] and archaea (*H. volcanii*) [56., 57., 58.]. The role of S-layer glycosylation remains mostly unclear. However, in some organisms, it has been implicated in the stabilization of the S-layer, maintenance of cell shape, adaptation to changing environmental conditions, formation of biofilms, formation of lubricating hydration layers, and modulation of a host immune response.

Protein lipidation is a further type of post-translational modification in which lipid moieties are covalently linked to proteins. It has been shown to be essential for the attachment of the *H. volcanii* SLP to the cytoplasmic membrane [39,40,42]. Finally, probably the rarest class of post-translational modification observed in SLPs is tyrosine phosphorylation. Thus far, it has been described only for the SLP of the bacterium *Aeromonas hydrophila* and is involved in lowering its isoelectric point (pI) [59].

## Structural Studies of S-layers

There is tremendous excitement in the S-layer field presently, owing to the recent elucidation of several high-resolution X-ray crystallography and electron cryomicroscopy (cryo-EM) structures, which are revolutionizing the field and advancing our understanding of S-layers. In the past, atomic models could not be produced routinely for S-layers due to difficulties associated with electron crystallography, which was the method of choice for studying S-layers [12,60]. In particular, for S-layers, nanobody-assisted crystallization [61] has allowed researchers to obtain 3D (three-dimensional) crystals of SLPs, leading to atomic structure solution using X-ray crystallography. The cryo-EM resolution revolution [62,63] has further intensified the ability of researchers to produce structures of SLPs. With the advent of powerful techniques such as electron cryotomography (cryo-ET) to solve macromolecular structures [64], structures of S-layers can now be solved directly in their cellular context.

**Figure 4:**
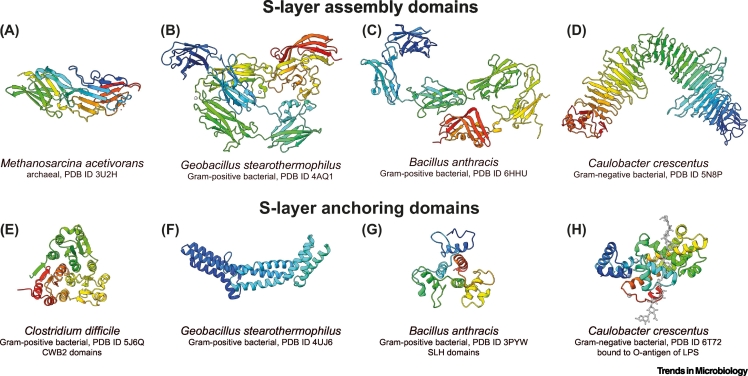
Structural Biology of Prokaryotic S-Layers. Atomic structures of surface-layer protein (SLP) domains resolved using X-ray crystallography and electron cryomicroscopy (cryo-EM). (A–D) Structures of lattice-forming assembly domains of SLPs. (E–H) Cell-anchoring SLP domains. All figures were made using publicly available atomic coordinates from the protein data bank (PDB) using UCSF ChimeraX [78]. The source organism and the PDB IDs of the structures are indicated below the atomic ribbon diagram colored as rainbow from N to the C terminus.

Structures of several S-layer lattice-forming domains have been resolved recently (Figure 4A–D). Taking advantage of the lattice-forming ability of SLPs, an archaeal SLP from *Methanosarcina acetivorans* [24] and a Gram-negative bacterial S-layer from *C. crescentus* [17] were solved using X-ray crystallography. This was made possible by the crystallization of the SLPs into a 3D crystal. In the case of the *C. crescentus* SLP, fortuitous cleavage of the S-layer anchoring domain allowed the S-layer lattice sheets to stack on top of each other [17], leading to the formation of 3D crystals. Nanobody-assisted crystallization circumvents this issue of 2D sheet formation entirely by disrupting the ability of the SLP to form an S-layer lattice. This approach allowed structure determination of the Gram-positive S-layer assembly domains from *G. stearothermophilus* [16] and *B. anthracis* [7]. These new S-layer assembly domain structures (Figure 4A–D) show that these domains are rich in β-strands and form tight lattice structures by initiating multiple contacts along the lattice [17,24].

In addition to lattice formation, SLPs must interact with prokaryotic envelopes to remain anchored to the cell and thereby perform their role at the cell surface. Recently, several structures of S-layer anchoring domains have been reported (Figure 4E–H). The structures of the three aforementioned Gram-positive bacterial anchoring domains, CWB2 [22], SLH [20], and *Geobacillus* SCWP-binding domains [21], have been solved with X-ray crystallography revealing predominantly α-helical proteins that interact directly with cell wall components. For Gram-negative bacteria, single-particle cryo-EM was used to solve the structure of the LPS binding domain of the *C. crescentus* SLP, revealing for the first time how the O-antigen is bound noncovalently to the S-layer [18]. No atomic structural data for archaeal anchoring domains are available thus far, and therefore, details of S-layer anchoring on archaeal cells remains poorly understood. Several different mechanisms of anchoring are probably at play in archaea depending on the species, and future structural biology research will be needed to understand in atomic detail how archaeal S-layers are anchored on cells.

**Figure 5:**
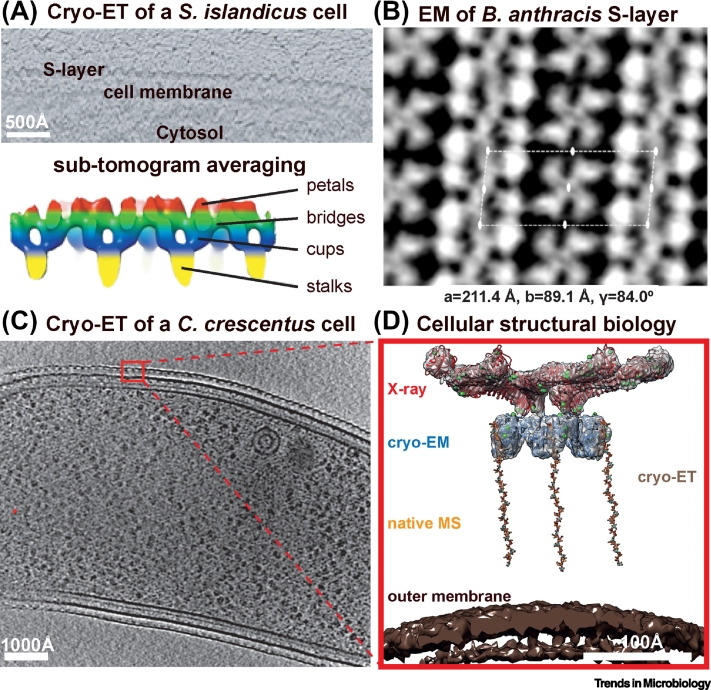
Structural Biology of Assembled S-layers. Examples of high-resolution electron cryomicroscopy (cryo-EM) and electron cryotomography (cryo-ET) analyses of assembled S-layers. (A) Cryo-ET and sub-tomogram averaging of the archaeal S-layer from *Sulfolobus islandicus*. Adapted with permission from Dr Bertram Daum [23]. (B) Cryo-EM projection image of the *Bacillus anthracis* Sap S-layer. Adapted with permission from Prof Han Remaut [7]. (C,D) Cryo-ET and sub-tomogram averaging structure of the S-layer from Gram-negative *Caulobacter crescentus* bacteria bound to the O-antigen of lipopolysaccharide (LPS) [18].

Despite recent progress in resolving structures of individual SLP domains, these structures in isolation can rarely explain the architecture and arrangement of the S-layer on cells. Cryo-ET has emerged as an extraordinary tool to solve structures of assembled S-layers *in vitro* or *in situ* on cells [65], providing unprecedented insight into S-layer structural biology. Cryo-ET and cryo-EM studies can now shed light on how S-layers are assembled on cells (Figure 5), applied to archaeal [23], Gram-positive [7], and Gram-negative bacterial S-layers [18]. Resolutions achievable in cryo-ET are not quite high enough currently; however, potent microscopy and image processing tools [66., 67., 68., 69.] are beginning to be applied to S-layer structural biology. Using such studies, the relative arrangements of the S-layer assembly and anchoring domains could be discovered in the archaeon *S. islandicus* [23]. In the Gram-negative bacterial S-layer of *C. crescentus*, in addition to the arrangement of the domains, density for the O-antigen of LPS could be observed, proving conclusively that the S-layer is tethered to the cell envelope through noncovalent interactions with the LPS [18].

## Common Themes in S-layer Assembly on Prokaryotic Cells

**Figure 6:**
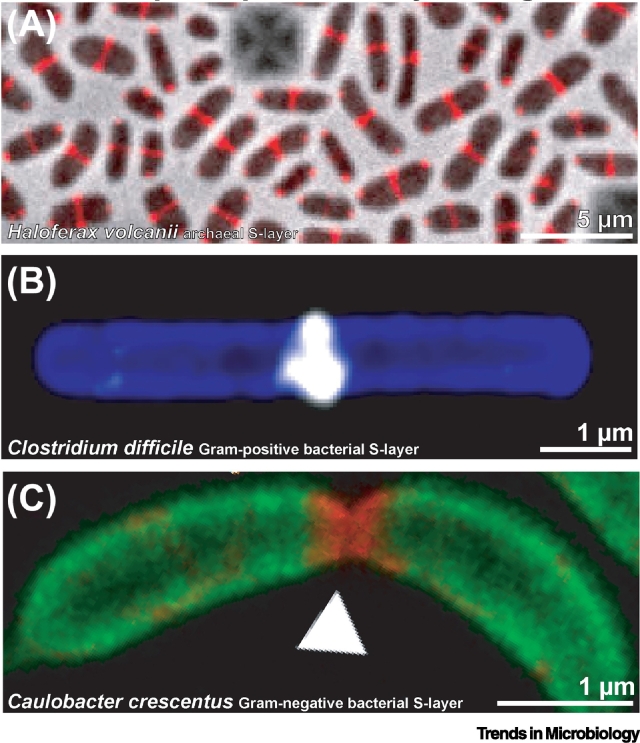
Mid-cell Surface-Layer Protein (SLP) Insertion Observed across Prokaryotes. (A) Fluorescently tagged SLPs were assembled at the mid-cell of *Haloferax volcanii* archaea [39]. (B) In Gram-positive *Clostridium difficile* bacteria, high-resolution imaging of S-layer assembly revealed similarly directed insertion of new SLP subunits at the mid-cell [71]. (C) Super-resolution imaging of SLP insertion in *Caulobacter crescentus* showed that most SLPs were inserted at the mid-cell, with some insertion observed at the cell poles [70].

Despite the variation in SLP sequences and structures discussed in this article, and despite the vastly different biochemical properties of envelopes in archaea, Gram-positive, and Gram-negative bacteria, some surprising common themes in S-layer biogenesis have been emerging in recent years [39,70,71]. Using real-time optical microscopy and a variety of strategies to fluorescently tag SLPs, the biogenesis of S-layers could be tracked, for the first time, at high-resolution in some archaea and bacteria, including *H. volcanii*, *C. crescentus*, *and C. difficile* (Fig. 6). These live imaging experiments showed that SLP insertion occurs predominantly at the mid-cell and is probably linked with the cell division and cell elongation machinery of prokaryotes [72]. Although this tantalizing observation has thus far only been reported in a small number of organisms, it perhaps reflects a common and widespread solution to a problem of needing to form a coat on a curved surface, along with the need to form breaks in the lattice for the insertion of new subunits. More research will need to be performed into the molecular cell biology of S-layer biogenesis, to reveal how deep these mechanistic principles are shared across prokaryotes (see Outstanding Questions).

## Concluding Remarks

With an increased focus on the structural and cell biology of S-layers, with an ever-increasing focus on archaeal biology [73], together with the immense potential of S-layers in nanotechnology [74,75], and the realization that S-layer biology is a fundamental property of prokaryotes, we anticipate an increased focus on this topic. Novel structural and cell biology techniques will aid our inquiries into S-layer biology, leading to more in-depth insights into these fascinating 2D arrays that assemble on prokaryotic cells with high co-cooperativity. We expect a much greater focus on the fundamental cell-biology mechanisms linking S-layer biogenesis to the prokaryotic cell cycle, and on using SLPs and S-layers to understand post-translational modifications and protein secretion across multiple domains of life.

Outstanding Questions The most important questions that will drive future research into S-layers are the reasons for similarities in S-layer assembly mechanisms and their regulation with respect to the cell cycle. What is the contribution of divergent and convergent evolution to this process? Can S-layer biology shed light on the evolution of life? Why is S-layer biogenesis coupled so tightly with the prokaryotic cell cycle, with new S-layer insertion predominantly at the mid-cell? Is this the most parsimonious solution to a complex cell biological problem? Armed with tools of modern structural and cell biology, the field is poised to answer these important questions that will not only shed light on a critical aspect of prokaryotic life but will have important implications in the synthesis of novel, biologically inspired nanomaterials.

Alt-text: Outstanding Questions
